# Recent Progress in Mass Spectrometry-Based Metabolomics in Major Depressive Disorder Research

**DOI:** 10.3390/molecules28217430

**Published:** 2023-11-04

**Authors:** Mingxia Liu, Wen Ma, Yi He, Zuoli Sun, Jian Yang

**Affiliations:** 1 Beijing Key Laboratory of Mental Disorders, National Clinical Research Center for Mental Disorders, National Center for Mental Disorders, Beijing Anding Hospital, Capital Medical University, Beijing 100088, China; liumingxia14@mails.ucas.ac.cn (M.L.);; 2 Advanced Innovation Center for Human Brain Protection, Capital Medical University, Beijing 100069, China; 3 State Key Laboratory of Natural and Biomimetic Drugs, School of Pharmaceutical Sciences, Peking University, Beijing 100191, China

**Keywords:** major depressive disorder, mass spectrometry, metabolomics, biomarkers

## Abstract

Major depressive disorder (MDD) is a serious mental illness with a heavy social burden, but its underlying molecular mechanisms remain unclear. Mass spectrometry (MS)-based metabolomics is providing new insights into the heterogeneous pathophysiology, diagnosis, treatment, and prognosis of MDD by revealing multi-parametric biomarker signatures at the metabolite level. In this comprehensive review, recent developments of MS-based metabolomics in MDD research are summarized from the perspective of analytical platforms (liquid chromatography-MS, gas chromatography-MS, supercritical fluid chromatography-MS, etc.), strategies (untargeted, targeted, and pseudotargeted metabolomics), key metabolite changes (monoamine neurotransmitters, amino acids, lipids, etc.), and antidepressant treatments (both western and traditional Chinese medicines). Depression sub-phenotypes, comorbid depression, and multi-omics approaches are also highlighted to stimulate further advances in MS-based metabolomics in the field of MDD research.

## 1. Introduction

Major depressive disorder (MDD) is a debilitating and widespread psychiatric illness characterized by enduring and substantial feelings of sadness, inferiority, and despair [1]. Notably, the World Health Organization has listed depression as the third leading cause of disease burden across the world and has predicted that the disease will rank first by 2030 [2]. However, due to the complicated pathogenesis of depression and the lack of pathophysiological biomarkers, the diagnosis and treatment of MDD using subjective evaluation and “trial-and-error” approaches often involve considerable error rates [3].

Metabolites are the downstream products of transcription and translation, and changes in those closest to a given phenotype can reflect many pathological or internal changes in biochemical pathways [4]. Metabolic disorders are considered to be an etiological factor in MDD, and metabolite analysis can certainly improve our understanding of the many pathological processes involved in MDD [5,6]. Metabolomics is the culmination of the cascade of “omics” technologies. It combines advanced analytical instrumentations with pattern recognition algorithms to reveal and monitor changes in metabolite profiles in subjects based on their disease status or response to medical or other interventions [7]. Advances in metabolomics have opened new avenues for exploring mechanisms related to MDD.

The main analytical platforms in metabolomics are nuclear magnetic resonance (NMR) and mass spectrometry (MS) [8]. NMR enables non-invasive analysis and relatively fast and straightforward metabolite annotation, but is less sensitive than MS. In-depth explanations and discussions of NMR-based metabolomics can be found in various excellent studies and reviews [9,10,11]. MS is widely used in metabolomics analyses. It combines rapidly developing separation technologies—primarily liquid chromatography (LC) and gas chromatography (GC)—to allow qualitative and quantitative analysis of multiple organic molecules in complex biological matrices (serum, plasma, urine, tissue, etc.) with high specificity, sensitivity, and throughput, and low sample consumption [12]. Given these advantages, the applications of MS in metabolomics research have grown exponentially in recent years.

This review focuses on advances in research into MDD using MS-based metabolomics. Common analytical procedures and key metabolic changes during pathogenesis and treatment are described, and current challenges and prospects are discussed with a view to enhancing research into this condition.

**Figure 1:**
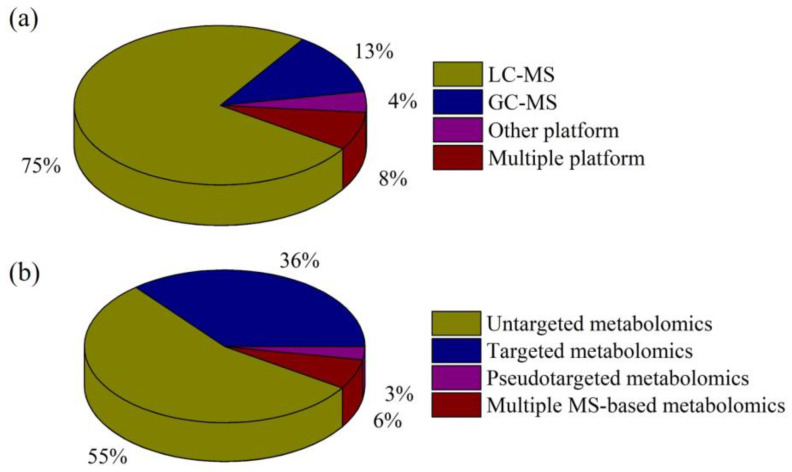
Percentages of reviewed, published studies utilizing various (**a**) MS-based analytical platforms and (**b**) metabolomics strategies.

A search of electronic literature bases from 2020 to August 2023 (PubMed [*n* = 170] and Web of Science [*n* = 192]) was conducted using the keywords “depression”, “metabolomics”, and “mass spectrometry”. After a preliminary review of these studies, articles in which the disease studied was not depression or the research method was not metabolomics were excluded. In addition, the reference lists of all identified studies were manually searched to identify any additional studies. Finally, 142 studies that met our criteria were identified, and these reports were reviewed. Figure 1 summarizes the numbers of studies in our review for the different MS-based analytical platforms and metabolomics strategies.

## 2. MS-Based Metabolomics Platforms in Depression Research

### 2.1. MS Platforms in Depression Research

Current state-of-the-art metabolomics technologies are mostly based on MS. Due to the need for measurement of isomers, isobars, and structurally similar analogs, chromatographic MS is preferred for metabolite profiling. LC-MS and GC-MS are the two primary platforms used in metabolomics research into depression, although other platforms, such as supercritical fluid chromatography-MS (SFC-MS) and capillary electrophoresis-MS (CE-MS), also play significant roles (Figure 1a).

#### 2.1.1. LC-MS

LC-MS is capable of detecting most compounds, including non-volatile and thermally labile metabolites, with or without derivatization and is, thus, the most frequently used platform in metabolomics analysis of depression. LC-MS analysis uses various types of columns, including reverse phase (RP-LC-MS; e.g., C18, C8, and C30 columns), normal phase (NP-LC-MS), and hydrophilic interaction (HILIC-LC-MS). RP and HILIC are mainly used for separation of weakly polar and polar compounds, respectively. Recently, an all-in-one-injection HILIC-MS/MS method was developed for the simultaneous determination of 20 purine and pyrimidine metabolites and used to show greatly disturbed purine metabolism in the serum and hippocampus of depressed mice [13]. Our group established a convenient LC-MS/MS method for the simultaneous measurement of 18 amino acid enantiomers using a conventional octadecylsilane RP column and chiral derivatization reagent. Significant differences in glycine, l-threonine, and d-methionine between late-life depression patients and controls were revealed by this method [14]. Sensitive Profiling ChemoSelective Derivatization Carboxylomics (SPCSDCarboxyl) was proposed by Zhou’s group in 2023 for the analysis of carboxylic acids using 5-(diisopropylamino) amylamine derivatization and ultra-performance LC-quadrupole time-of-flight MS (UHPLC-Q-TOF/MS) [15]. Two hundred and eight metabolites were identified in the serum of depressed patients using SPCSDCarboxyl, and a combination of proline, 1-pyrroline-5-carboxylate, and glutamic acid could distinguish between patients and healthy controls. Mocking et al. measured 399 metabolites in patients with recurrent MDD using an established LC-MS/MS platform, and 80% of the recurring metabolic predictors belonged to the phospholipid, sphingomyelin, glycosphingolipid, eicosanoid, microbiome, or purine pathways [16].

#### 2.1.2. GC-MS

GC-MS is suitable for the analysis of volatile organic compounds (VOCs), although derivatization is required to increase the thermal stability and volatility of non-volatile compounds and to reduce their polarity. Due to the high reproducibility of electron ionization in MS, GC-MS can utilize many mass spectra libraries, which enables relatively easy identification of peaks. Several studies have shown that urinary metabolite biomarkers identified by GC-MS can identify post-stroke depression (PSD) in stroke survivors [17]. A biomarker panel consisting of glyceric acid, tyrosine, and azelaic acid was identified in middle-aged and elderly patients with PSD [18,19]. Solid-phase microextraction and GC-MS were used to analyze urinary VOCs and semi-VOCs in patients with late-life major depressive and anxiety disorders. The combined indicators dimethylsulfone, phenethyl isothiocyanate, hexanoic acid, texanol, and texanol isomers showed excellent performance in evaluating MDD and/or agoraphobia in the elderly [20].

#### 2.1.3. Other Chromatography-MS Platforms

Interest in SFC-MS in depression research has grown in recent years due to its excellent separation capabilities and environmental friendliness. It shows remarkable performance in the analysis of lipids. In-line supercritical fluid extraction coupled with SFC-MS/MS method was used to rapidly separate 23 inflammation-related lipids in brain tissue of depressed rats within 15 min. Six pro-inflammatory lipids increased in depressed rats, while six anti-inflammatory lipids decreased [21]. Analysis of VOCs in exhaled breath using proton-transfer-reaction MS (PTR-MS) is a hot topic in the field of depression research, given its advantageous real-time, in-line, and non-invasive attributes. Lueno et al. conducted the first PTR-MS study of the differences in VOCs in exhaled breath in MDD patients and healthy controls. There were significant differences in several masses between the groups, with *m/z* = 69, 74, 93, and 94 being identified as potential high-accuracy biomarkers [22]. This group then applied breathomics (one of the newest branches of metabolomics) using untargeted PTR-MS to explore changes in biochemical patterns and metabolic pathways related to MDD. A total of 23 differential exhaled metabolites were significantly altered in MDD patients, and these were mapped to five metabolic pathways [23]. Recently, an interesting study from Frodl’s group used PTR-MS to analyze gut–brain axis VOCs and distinguish between schizophrenia, MDD, and healthy controls [24]. CE-MS is a powerful tool that combines the high separation capability and low sample consumption of CE with the identification capabilities of MS. Okamoto et al. used CE and Fourier transform MS to identify differential patterns of serum metabolites in MDD patients with and without type 2 diabetes mellitus, indicating that this comorbidity can affect metabolic pathways and alter the distribution of serum metabolites in MDD patients [25].

#### 2.1.4. Combined Chromatography-MS Platforms

A single analytical technique cannot encompass all metabolites, given their wide-ranging physicochemical properties and broad concentration ranges. For example, Xie’s group used GC-MS to characterize differential metabolites in the olfactory bulb (OB) of rats with chronic unpredictable mild stress (CUMS). Disruption of lipid and purine metabolisms was demonstrated, which may be related to dysfunction of the OB [26]. Subsequently, Zhou’s group used LC-MS to investigate metabolic changes in the OB of mice and, in contrast to the previous GC-MS results, demonstrated disruption of the tryptophan-5-hydroxytryptamine pathway [27]. These findings show that different analytical techniques can highlight different metabolic perspectives, and it is necessary to adopt multiple chromatography-MS platforms in the search for new depression biomarkers and molecular mechanisms. A combination of GC-MS and LC-MS/MS was used to analyze metabolite profiles in plasma, urine, and cerebrospinal fluid (CSF) of patients with treatment-refractory depression and suicidal behavior [28]. A significant proportion of patients showed treatable abnormalities, while no healthy controls exhibited metabolic abnormalities. A metabolome-wide association study using two separate UHPLC-MS/MS injections and one GC/MS injection of each sample found that the level of lauroylcarnitine in serum was decreased in patients with depression, which may indicate fatty acid oxidation and/or mitochondrial dysfunction in depression [29].

### 2.2. Metabolomics Strategies in Depression Research

Metabolomics is a branch of “omics” technology focusing on high-throughput identification and quantification of small molecule metabolites (<1500 Da). It can describe specific multi-parameter characteristics of the heterogeneous pathophysiological mechanisms underlying depression. There are three main MS-based metabolomics approaches in depression research: untargeted, targeted, and pseudotargeted analyses (Figure 1b).

#### 2.2.1. Untargeted Metabolomics

Untargeted methods are typically used in metabolomics studies for the detection and discovery of small organic compounds, with high-resolution MS (HRMS) using Orbitrap or Q-TOF instruments providing full-scan information, accurate masses, and tandem MS details of the metabolites. Although untargeted metabolomics suffers from high complexity, poor repeatability, and limited linear range, it remains the first choice for the metabolite discovery stage because it is unbiased and has high coverage. Jiao et al. used the classic untargeted metabolomics technique (UHPLC-Q-TOF-MS) to investigate the antidepressant-like effects of Jiaotaiwan on rats [30]. Changes in the metabolite profile of rat serum before and after administration were analyzed using multiple statistical approaches. The most important biomarkers associated with depression were identified via principal component analysis, partial least squares discriminant analysis, and heatmap analysis. Pathway analysis then revealed that the therapeutic effect of Jiaotaiwan on depression may involve the regulation of amino acid, glycerophospholipid, and energy metabolisms. Untargeted metabolomics was also used to identify O-acetyl-l-carnitine, l-aspartic acid, fumarate, and alanine as peripheral biomarkers in patients with MDD [31]. To clarify the metabolites involved in specific pathways, a stable isotope-resolved metabolomics method was developed and applied in depression research for the first time by Qin’s group [32]. The stable isotope tracer ^13^C_6_-glucose was prepared and introduced into a CUMS rat model, and labeled metabolites were detected by LC-MS using HILIC and T3 chromatography columns. Twenty-eight of the 78 labeled metabolites related to energy metabolism in the model group differed significantly from the control group.

#### 2.2.2. Targeted Metabolomics

Targeted metabolomics based on triple-quadrupole MS (TQMS) is generally used in the verification phase to confirm the identity of, and to quantify, compounds of interest. When using multiple reaction monitoring (MRM) mode, targeted analysis is characterized by high sensitivity, strong specificity, good repeatability, and wide linear range, but it is limited by its relatively narrow coverage of metabolites. However, continuous development of ionization efficiency, scanning rate, and other parameters has enabled the simultaneous analysis of dozens to hundreds of metabolites by TQMS. Energy-related metabolites, carnitine, amino acids, and biogenic amines were quantified in the ventral hippocampus of rats with chronic mild stress (CMS) using LC-MS/MS. Glycolysis and the tricarboxylic acid cycle were particularly involved in defining vulnerability to stress [33]. To avoid the addition of internal standards and corresponding analogs, Chen et al. developed a targeted metabolomics method involving relative quantification based on HILIC-MS/MS and the quality control-based random forest signal correction algorithm [34]. Nineteen metabolites were simultaneously determined in the serum of MDD patients, the changes in urocanic acid being reported for the first time.

#### 2.2.3. Pseudotargeted Metabolomics

The recently developed pseudotargeted metabolomics approach combines the benefits of targeted and untargeted analyses. By extracting MRM transitions from biological samples, pseudotargeted profiling offers higher coverage of metabolites than targeted profiling. Furthermore, the use of selected ion monitoring (SRM) mode gives pseudotargeted profiling a wider linear range and better data quality than untargeted profiling. However, some limitations of pseudotargeted metabolomics still need to be addressed. For example, a combination of HRMS and TQMS is usually required, some detected metabolites cannot be identified, and it is only semi-quantitative [35]. There are many applications of targeted and untargeted metabolomics in studies of depression, but only a few studies have used pseudotargeted methods. In 2020, Yang et al. described a segment data-dependent acquisition (SDDA)-based pseudotargeted approach for analysis of depressed rats treated with liquiritin [36]. A total of 502 MRM transitions were detected, and five metabolic pathways were found to be related to depression. This same research group subsequently developed comprehensive pseudotargeted lipidomics methods based on SDDA and two- or three-phase liquid extraction to elucidate the differential lipids related to depression. Broadening the lipid coverage and addressing analyte co-elution enabled 53 and 61 differential variables to be identified in the plasma of depressed rats in these studies [37,38]. Yang et al. also described a green and efficient ultra-high performance supercritical fluid chromatography-MS (UHPSFC-MS/MS)-based pseudotargeted lipidomics method that detected 758 lipids within 8 min [39]. This method had a shorter analytical runtime, narrower peaks, higher sensitivity, and better separation of lipid isomers than the UHPLC-MS/MS-based pseudotargeted method. Applications of the pseudotargeted metabolomics approach in depression research are still in their infancy but show great potential.

#### 2.2.4. Combined Metabolomics Strategies

Increasing attempts are being made to determine the complete metabolite profile for depression by combining multiple MS-based metabolomic approaches. Untargeted methods are often used as an initial screening assay in clinical biomarker discovery studies, with only those metabolites showing significant differences being confirmed using targeted, quantitative assays. Lee et al. profiled serum metabolites using an untargeted method, identifying 14 metabolites with differences between MDD and control groups [40]. The efficacy of endogenous acetylcarnitine for the diagnosis of depression and determination of remission status was then confirmed using a targeted SRM approach. Similarly, Wang et al. used untargeted serum metabolomics and pathway analysis to show that abnormal amino acid metabolism in mice with chronic social defeat stress (CSDS) is related to their abnormal behavior, and the reduction in leucine revealed by targeted metabolomics is specifically and positively related to the social interaction rate [41]. The antidepressant mechanism of the Chaihu–Baishao herb pair was investigated using combined untargeted and targeted analyses [42]. Twenty-one metabolic pathways that were synergistically regulated by Chaihu–Baishao were identified via cortex metabolomics based on UPLC-Q-Orbitrap/MS, and the crucial impact on the purine metabolism pathway was quantitatively confirmed by UPLC-MS/MS in MRM mode.

## 3. Key Metabolic Changes in Depression

Advances in MS-based metabolomics techniques have been crucial in driving the progress of research into depression. Recent applications of MS-based metabolomics in depression biomarker discovery and elucidation of pathogenic mechanisms are summarized below.

### 3.1. Monoamine Neurotransmitters

The “monoamine hypothesis” is important in the study of depression, and the development of the majority of clinical antidepressants has been based on monoamine neurotransmitters [43]. Although considerable progress has been made in this area, the underlying mechanisms remain unclear and treatments are increasingly controversial. Monoamine neurotransmitters can interact with other metabolic pathways in depression. The “monoamine (5-HT)-Glutamate/GABA long neural circuit”, proposed by Li, holds the view that monoaminergic and non-monoaminergic mechanisms form a long neural circuit that mediates rapid antidepressant effects [44]. Li et al., using LC-MS/MS, studied changes in neurotransmitters and their related metabolites in GABAergic, serotonergic, and catecholaminergic pathways in the nucleus accumbens of CUMS-induced anhedonia-like rats [45]. The level of 5-hydroxytryptamine in anhedonia-susceptible rats increased, while dopamine did not change significantly. Xu et al. found that gut microbiota (GM) can activate monoamines via stimulating the enteroendocrine cells to produce 5-hydroxytryptamine, dopamine, and norepinephrine, which can affect the central nervous system. The brain in turn can regulate gastrointestinal functions through the neuro-immune-endocrine system [46]. Using LC-MS/MS, Zhong’s group showed that *Morinda officinalis* oligosaccharides alleviated depression via the tryptophan-5-hydroxytryptophan-serotonin metabolic pathway in the GM [47]. In addition, monoamine neurotransmitters are intertwined with numerous new depression pathways, such as inflammation, oxidative stress, neurotrophins, and neurogenesis. In-depth explanation and discussion can refer to some excellent works and reviews [5,43].

### 3.2. Amino Acids

Amino acids and their metabolites are fundamental substrates and regulators in many metabolic pathways and some have been identified as biomarkers of depression. Untargeted GC-MS identified significant changes in l-alanine, l-glutamic acid, glycine, l-methionine, l-phenylalanine, l-valine, l-isoleucine, and l-norleucine in the main stress-targeted tissues of CUMS-induced mice [48]. High levels of glutamic acid, aspartic acid, and glycine and low levels of 3-hydroxykynurenine were quantified by LC-MS in serum of MDD patients, and the levels of glutamic acid and phenylalanine correlated with the severity of depression [49]. Significant negative associations of the branched-chain amino acids valine and leucine with depression were identified using untargeted metabolomics [50]. Increased glutamate, decreased dopamine, and altered trends in γ-aminobutyric acid in the habenula of CUMS-susceptible and -resilient rats were identified using LC-MS/MS [51].

Disruption of the tryptophan pathway plays a crucial role in MDD. Tryptophan is metabolized alongside the kynurenine, serotonin, and microbial pathways. Brum et al. found that levels of all tryptophan catabolites were reduced in the plasma of patients with MDD, bipolar depression (BD), and schizophrenia (SCZ), but these metabolites could not be used to distinguish between the disorders [52]. A similar conclusion was also reached by Liu et al. [53]. Yun et al. studied the relationship between the tryptophan–kynurenine pathway and the painful physical symptoms of MDD [54]. Patients with such symptoms exhibited higher kynurenine, quinolinic acid, and kynurenine/tryptophan ratios than those without. Tryptophan metabolism is central to communication between the GM and the brain in depression [55]. LC-MS/MS showed that kynurenine and 3-hydroxycaninuric acid increased significantly along the gut–brain axis of depressive-like rats subjected to chronic restraint stress (CRS) [56]. The tryptophan–kynurenine pathway is also linked to the inflammatory state of patients with MDD [57]. Haroon et al. analyzed kynurenine pathway metabolites and inflammatory markers in the plasma and CSF of depressed patients [58]. Kynurenine and kynurenine/tryptophan in plasma, and kynurenine, kynurenic acid, and quinolinic acid in CSF were closely related to plasma tumor necrosis factor. Pau et al. replicated and expanded upon these findings by evaluating more metabolites and suggesting that the levels of some peripheral kynurenine pathway metabolites might serve as proxies for central kynurenine pathway metabolites in patients with MDD [59]. Zheng et al. also found that C-reactive protein and kynurenic acid/quinolinic acid are independently associated with white matter integrity in MDD [60]. Some studies indicate that therapy can affect tryptophan metabolism. Tateishi et al. reported that levels of kynurenine, kynurenic acid, and kynurenine/tryptophan ratio in plasma of patients with treatment-resistant depression were unchanged after repetitive transcranial magnetic stimulation treatment [61]; however, Ryan et al. reported that the kynurenic acid pathway was mobilized by electroconvulsive therapy [62].

### 3.3. Lipids

Lipids are a broad class of biomolecules with essential roles in many cellular processes, including molecular signal transduction, energy storage, and cell membrane formation. Advanced MS-based lipidomics methods have deepened our understanding of the lipidome in the central and peripheral nervous systems and its associations with depression [6]. Miao et al. identified lipid networks associated with the risk of depression using untargeted LC-MS lipid analysis [63]. For example, lower levels of sphingomyelins and glycerophospholipids and higher levels of lysophospholipids were associated with the incidence and/or prevalence of depression. An LC-MS lipidomics study identified 13 differentially expressed lipids in the plasma of adult female MDD and BD patients and could distinguish between these conditions with medium confidence (area under the curve [AUC] was 0.860) [64]. Similarly, a panel of 111 lipid species was capable of distinguishing SCZ from MDD (AUC = 0.920) [65]. Glycerophospholipids are critical components of neuronal membranes and eukaryote cellular membranes. LC-MS lipid metabolite profiling in the hippocampus of PSD rats showed 50 key metabolites were reduced, and these were mainly involved in glycerophospholipid metabolism (particularly cardiolipin metabolism) [66]. Glycerophospholipid metabolism was also associated with the pathogenesis of PSD in humans [67,68]. Various lipidomics studies have confirmed that peripheral and central glycerophospholipid metabolism disorders are involved in the pathogenesis of depression via the microbiome–gut–brain axis [69,70,71,72]. Jiang et al. used UHPLC-Q-TOF-MS to investigate plasma metabolite biomarkers in young MDD patients and identified phosphatidylcholine as a female-specific biomarker (AUC = 0.957) [73]. Schumacher et al. found that ceramide concentration in the plasma of MDD patients correlated with the severity of MDD, and neutralization of ceramides abrogated depressive behavior in mice [74]. Untargeted UHPLC-MS metabolomics revealed that phosphatidylserine (16:0/16:1) and phosphatidic acid (18:1/18:0) were significantly increased in plasma of MDD patients [75].

### 3.4. Energy Metabolism

Many studies have shown that energy metabolism is impaired in patients with depression. This may point towards new treatments for the condition. Most of the body’s energy comes from the tricarboxylic acid cycle, oxidative phosphorylation, and glycolysis [76]. Wang et al. demonstrated, using metabolomics, that the tricarboxylic acid cycle was inhibited in mice exposed to CSDS and in patients with first-episode depression [77]. The altered metabolism of acylcarnitines may link mitochondrial dysfunction to depression via impairment of fatty acid β-oxidation [78]. Lower levels of acetyl-l-carnitine and medium- and long-chain acylcarnitines and higher levels of l-carnitine and l-carnitine/acetyl-l-carnitine ratio were found in the plasma of depressed patients, but these differences disappeared after treatment [79,80]. Acylcarnitine profiles also help to distinguish different phenotypic subtypes of MDD, such as core depression, anxious depression, and neurovegetative symptoms of melancholia [81]. Given that glycogen is the main energy source for most higher organisms, Qin’s group used stable isotope-resolved metabolomics with a ^13^C_6_-glucose tracer to reveal the blockage of the tricarboxylic acid cycle and abnormal activation of gluconeogenesis in rats with CUMS and in corticosteroid-induced PC12 cells [82,83,84,85,86].

### 3.5. Gut Microbiota and Metabolomics

The relationship between the GM and depression is a particular focus of psychobiology research, but the underlying molecular mechanisms remain unclear [87]. A combination of 16S rRNA gene sequencing and MS-based metabolomics is often used to investigate these GM mechanisms in patients with depression and in CUMS, CSDS, and CRS mouse models [88]. Growing evidence from this toolkit of clinical studies and animal models suggests that GM compositions (e.g., the phylum Firmicutes and genera *Bacteroides* and *Lactobacillus*) and related metabolites (e.g., short-chain fatty acids and tryptophan metabolism) are disordered in depression along the brain–gut–microbiota axis. For example, Xie et al. found that two crucial tryptophan metabolism-related metabolites (tryptophan and 5-hydroxytryptophan) were reduced in the feces of CSDS mice, and these compounds were associated with *Lactobacillus* [89]. Zhang et al. showed that *Bacteroides* species enriched in the GM of MDD patients had differing effects on the susceptibility to depressive behaviors [90]. This was partly explained by the different changes in tryptophan pathway metabolites and neurotransmitters along the gut–brain axis. The relationship between microbial metabolites in feces and neurotransmitters in the prefrontal cortex of depressed mice was also explored using targeted metabolomics [91]. This suggested that the disruption of microbial metabolites may affect prefrontal cortex neurotransmitter levels, leading to depressive episodes. This same phenomenon—simultaneous changes in brain and gut metabolism in CUMS rats—was also observed by Hu et al. [92]. Our group used whole-genome shotgun metagenomic and untargeted metabolomic methods to identify disturbed microbial genes (in *Bacteroides*, *Blautia*, and *Eubacterium*) and fecal metabolites (γ-aminobutyrate, phenylalanine, and tryptophan) in MDD patients [93]. The antidepressant effect of chenodeoxycholic acid regulated by *Blautia* and *Eubacterium* has also been studied [94]. Table 1 summarizes the GM-related metabolites that have been reported to be associated with depression.

## 4. Metabolomics in Antidepressant Treatment Response

Given the phenotypic complexity of patients’ responses to antidepressants, clinical symptoms and “trial-and-error” approaches are insufficient to guide treatment selection for individual patients. Pharmacotherapy is generally the first-choice treatment for MDD. Crucially, the metabolic status of MDD patients exhibiting a response to pharmacotherapy (including remission) appears to differ from non-responsive patients [106]. Pharmacometabolomics (the application of metabolomics in the study of drug effects) has been used to map the effects of antidepressants on metabolite profiles and has provided new insights into the mechanisms of action of various therapies. Some of the studies that have evaluated metabolite changes in animal models and clinical patients following antidepressant treatment are summarized in Table 2. Pharmacological medications have alleviated abnormalities of amino acid, energy, and lipid metabolisms and GM-derived metabolites induced by depression.

### 4.1. Western Medicines

Several notable western medicines have contributed significantly to the management of depression, although their mechanisms of action are not fully understood. Escitalopram is one example. It is a commonly used antidepressant of the selective serotonin reuptake inhibitor (SSRI) class, but the response varies between individuals. The mechanism of citalopram/escitalopram was studied using metabolomics targeted at 180 metabolites, and changes in the profiles of acylcarnitine, lipids, and amino acids indicated that mitochondrial energetics and lipid membrane remodeling are implicated in the SSRI treatment response [107]. Recently, our group conducted an LC-MS/MS study on the relationship between plasma oxysterol levels and the effectiveness of escitalopram antidepressant treatment. Oxysterols, especially 27-hydroxycholesterol, decreased in responders and increased in non-responders following escitalopram treatment. This suggests that 27-hydroxycholesterol has potential as an escitalopram response indicator during MDD treatment [108]. We also explored the role of the GM in determining escitalopram treatment efficacy in MDD patients [109]. Such microbiota-centered perspective could potentially improve antidepressant efficacy in clinical practice. The antidepressant effects of ketamine have received increasing attention since the United States Food and Drug Administration approved (S)-ketamine nasal spray in March 2019 [151,152]. Zhou et al. analyzed changes in lipid compositions in mice with induced chronic variable stress (CVS) and found that disruption of sphingolipids, glycerolipids, and fatty acyls was partially corrected by administration of (S)-ketamine [113]. MS-based metabolomics has been used to analyze the efficacy of various synthetic antidepressants and has made a significant contribution to improving the treatment of depression.

### 4.2. Traditional Chinese Medicines

Traditional Chinese medicines (TCMs) exhibiting desirable antidepressive effects have gradually attracted more attention because of their strong safety profiles. However, due to the multi-component, multi-target, and multi-channel nature of TCMs, elucidation of their mechanisms of action is challenging. MS-based metabolomics provides a new way to elucidate these mechanisms holistically [153]. For the first time, in 2021, a combination of pharmacodynamics and urine metabolomics based on UPLC-Q-TOF-MS was used to investigate the antidepressant effect of *Millettia speciosa* Champ [154]. l-isoleucine, sebacic acid, and allantoin were identified as potential pharmacodynamic biomarkers related to the efficacy of this TCM. Similarly, LC-MS-based metabolomics of peripheral blood mononuclear cells (PBMCs) was used to investigate the antidepressant mechanism of Chaigui granules [115]. Their antidepressant effects were attributed to improved immune function and regulation of the purine metabolic pathway in PBMCs. The metabolomics analysis of TCMs in Table 2 exhibits a systemic metabolic shift in amino acids (such as alanine, aspartate, glutamate, tryptophan, etc.), organic acids (oxalic acids, stearic acids, bile acid, etc.), and purine, phospholipid, etc. These differential metabolites are mainly involved in amino acid metabolism, lipid metabolism, energy metabolism, gut microbiota metabolism, etc. Such integration of metabolomics with other analytical strategies has provided new insights into the mechanisms of many TCMs and promoted their use as modern treatments for depression.

### 4.3. Other Treatments

Given the high proportion of refractory or treatment-resistant cases of depression, there is an urgent need for the development of new antidepressants. Metabolomics is an effective strategy in this field. l-theanine is a bioactive component of green tea and a food additive with health benefits. Zhu et al. systematically explored the antidepressant effects of l-theanine in a CUMS rat model using LC-MS/MS and enzyme-linked immunosorbent assay (ELISA) techniques [139]. Untargeted UPLC-Q-TOF-MS highlighted 28 metabolites that changed significantly during l-theanine treatment, while targeted HILIC-MS/MS identified these key amino acids and neurotransmitters and, consequently, their related pathways. By clarifying these preventive mechanisms, this study laid a foundation for the use of l-theanine in the treatment of children and adolescents with depression. Some probiotics also exhibit antidepressant effects and have fewer side effects, have less of an associated stigma, and are less addictive than conventional antidepressants [155]. The therapeutic effect of bifid triple viable probiotic capsules was evaluated in a CUMS rat model, and untargeted metabolomics revealed that the observed reduction in depression-like behavior may be related to endothelin-1 or CREB signaling [143].

## 5. Perspectives and Conclusions

MDD is a highly heterogeneous condition, but the use of metabolomics to identify specific biological characteristics of clinical sub-phenotypes is expected to improve personalized diagnostic capabilities. Brydges et al. used three metabolomics platforms to evaluate the correlation between metabolomic markers and three symptom dimensions of MDD (melancholic, anxious distress, and immunometabolic) [156]. These symptoms exhibited specific and minimally overlapping metabolomic signatures, suggesting that the multifaceted disruption of the delicate balance between the GM, dietary lipids, and host lipid metabolism may be a cause of specific MDD symptoms. It is clear that further detailed MS metabolomics studies of the various subtypes of depression are likely to improve clinical diagnosis.

In addition to subtypes of depression, increasing attention has been paid to metabolomics-based research of comorbid depression. Investigation of comorbid depression in mice under social fear conditions suggested that changes in sphingolipid metabolism in the brain may be related to the short- and long-term pathophysiology of social anxiety disorder [157]. The effects and mechanisms of Jiaotaiwan treatment of diabetes mellitus accompanied by depression, and of albiflorin and paeoniflorin in the treatment of cancer-related depression, have been evaluated using MS-based metabolomics, providing greater understanding of the mechanisms of antidepressant therapies [158,159]. Metabolomics is expected to be increasingly used in research into various diseases complicated by depression.

Due to the complexity of the pathogenesis of MDD, the integration of metabolomics with other “omics” technologies is becoming increasingly necessary. Recent studies have combined genomics and metabolomics to characterize various aspects of early- and adult-onset MDD, including adult MDD suicide attempts [160,161,162,163]. Integrated proteomics and metabolomics were used to explore antidepressant treatments in animal models and MDD patients [164,165]. Multi-omics methods will improve our understanding and treatment of MDD and enhance prevention strategies, enabling the considerable advancement of precision medicine [166].

Recent advances in MS-based metabolomics platforms have facilitated a more intensive study of depression. This review summarizes the main findings of the most recent studies in this field focusing on the applied platforms (LC-MS, GC-MS, SFC-MS, etc.) and strategies (untargeted, targeted, and pseudotargeted approaches). Key metabolic changes (in monoamine neurotransmitters, amino acids, lipids, energy metabolism, and GM-related metabolism) and the application of metabolomics in antidepressant treatments in western medicines and TCMs are also reviewed. Depression sub-phenotypes, comorbid depression, and multi-omics approaches are also discussed. We expect this review to stimulate new developments in MS-based metabolomics in the field of depression research.

## Figures and Tables

**Table 1:** Examples of metabolites associated with gut microbiota that have been reported to be associated with depression.

Gut Microbiome Profiling Method	Gut Microbiota	Metabolomics Method	Metabolic Pathway	Subject/Sample Type	Reference
16S rRNA gene sequencing	Phylum Firmicutes and genus *Lactobacillus*	Targeted metabolomics/UHPLC-MS/MS	Tryptophan metabolism	Mice (CSDS)/feces and hippocampus	[89]
16S rRNA gene sequencing and metagenomic analysis	*Lachnospiraceae*	Untargeted metabolomics/UPLC-Q-TOF-MS and targeted metabolomics/UPLC-MS/MS	Glycerophospholipid metabolism and γ-aminobutyric acid	Mice (CUMS)/feces, liver, and hippocampus	[72]
16S rRNA gene sequencing and metagenomic analysis	Phylum Firmicutes	Untargeted metabolomics/UPLC-Q-TOF-MS and targeted metabolomics/UPLC-MS/MS	Glycerophospholipid metabolism, tryptophan pathway, and short-chain fatty acids	Mice (CRS)/feces, serum, and hippocampus	[71]
16S rRNA gene sequencing	Phylum Firmicutes	Untargeted metabolomics/UPLC-Q-TOF-MS	Inflammation-related metabolites	MDD patients/serum and feces	[95]
16S rRNA gene sequencing	Phylum Firmicutes	Untargeted metabolomics/GC-MS and LC-MS	Glycerophospholipid metabolism	Cynomolgus macaque of depression/feces, peripheral, and brain tissue	[69]
16S rRNA gene sequencing	Genus *Allobaculum* and family *Ruminococcaceae*	Targeted metabolomics/LC-MS/MS and GC-MS	Acetic acid, propionic acid, pentanoic acid, norepinephrine, 5-hydroxy indole acetic acid, and 5-hydroxy tryptamine	Mice (CRS)/feces and hypothalamus	[96]
16S rRNA gene sequencing	Ten genera (most of them belonged to phylum Firmicutes)	Targeted metabolomics/GC-MS and untargeted metabolomics/LC-Q-Orbitrap/MS	Short chain fatty acids	Rats (PSD)/feces and prefrontal cortex	[97]
16S rRNA gene sequencing	Phylum Firmicutes, genus *Blautia*, and *Streptococcus*	Untargeted metabolomics/GC-MS	Lipid metabolism	Rats (PSD)/feces	[98]
16S rRNA gene sequencing	*Actinobacteria* and *Bacteroidetes*	Untargeted metabolomics/LC-Q-Orbitrap/MS and GC-MS	Glycerophospholipids	Mice (CSDS)/feces and prefrontal cortex	[70]
Whole-genome shotgun metagenomic	Genus *Bacteroides*, genera *Blautia*, and *Eubacterium*	Untargeted metabolomics/GC-MS	Amino acid metabolism (γ-aminobutyrate, phenylalanine, and tryptophan)	MDD patients/feces	[93]
Viral metagenomics	*Microviridae*, *Podoviridae*, and *Siphoviridae*	Targeted metabolomics/UPLC-MS/MS	Tryptophan metabolism	Mice (CRS)/feces	[99]
16S rDNA amplification sequencing	Deferribacteres, Proteobacteria, Verrucomicrobia, Actinobacteria, *Desulfovibrio*, *Clostridium_IV*, *Helicobacter*, *Pseudoflavonifractor*, and *Akkermansia*	Untargeted metabolomics/LC-MS/MS	Lipid metabolites, glycerophospholipid metabolism Pathway, and the retrograde endocannabinoid signaling pathway	Atherosclerosis co-depression mice/feces	[100]
16S rRNA gene sequencing	*Turicibacteraceae*, *Turicibacterales*, and *Turicibacter*	Targeted metabolomics/UPLC-MS/MS	Bile acids metabolism	MDD patients/blood and feces	[101]
Metagenomics sequencing	*Ruminococcus bromii*, *Lactococcus chungangensis*, and *Streptococcus* *gallolyticus*	Targeted metabolomics/HPLC-MS/MS	Lipid, vitamin, and carbohydrate metabolism	MDD patients/blood and feces	[102]
16S rRNA gene sequencing	*Bacteroides*	Untargeted metabolomics/UPLC-Q-TOF-MS and targeted metabolomics/UPLC-MS/MS	Tryptophan pathway metabolites and neurotransmitters	MDD patients/feces, serum, and tissue samples	[90]
16S rRNA gene sequencing	Phylum Firmicute, *Bacteroidetes*, genus *Faecalibacterium*, *Roseburia*, *Subdoligranulum*, and *Agathobacter*	Untargeted metabolomics/UPLC-Q-TOF-MS	Alpha-linolenic acid metabolism, biosynthesis of unsaturated fatty acids, ATP-binding cassette transporters, and bile secretion	Systemic lupus erythematosus patients with depression/feces	[103]
16S rRNA gene sequencing	*Streptococcus*, *Phascolarctobacterium*, *Akkermansia*, *Coprococcus*, and *Streptococcus*	Targeted metabolomics/LC-MS/MS	Indole-3- carboxyaldehyde	MDD patients/feces	[104]
16S ribosomal RNA gene sequencing	Family *Lachnospiraceae*, *Muribaculaceae*, and *Oscillospiraceae*	Untargeted metabolomics/LC-Q-Orbitrap/MS	Lipid and amino acid metabolism	Rats (CUMS, CRS, SD, and LH)/feces	[88]
16S rRNA gene sequencing	*Alistipes indistinctus*, *Bacteroides ovatus*, and *Alistipes senegalensis*	Untargeted metabolomics/LC-Q-Orbitrap/MS	D-pinitol, indoxyl sulfate, trimethylaminen-oxide, and 3 alpha, 7 alpha-dihydroxy-12-oxocholanoic acid	Rats (CUMS)/feces	[105]

Abbreviations: major depressive disorder (MDD), chronic social-defeat stress (CSDS), chronic unpredictable mild stress (CUMS), chronic restraint stress (CRS), post-stroke depression (PSD), social defeat (SD), and learned helplessness (LH).

**Table 2:** Applications of metabolomics in the analysis of treatments for depressive disorders.

Treatment		Subject/Sample Type	Analytical Platform	Metabolic Pathway	Reference
Western medicine	Citalopram, escitalopram	MDD patients/plasma	Targeted metabolomics/LC-MS/MS and flow-injection analysis-MS/MS	Mitochondrial energetics (acylcarnitine metabolism, transport, and β-oxidation) and lipid membrane remodeling	[107]
	Escitalopram	MDD patients/plasma	Targeted metabolomics/LC-MS/MS	Oxysterols	[108]
	Escitalopram	MDD patients/plasma and feces	Untargeted metabolomics/GC-MS	Amino acids and fatty acids	[109]
	Clomipramine	Rats with ultrasound model of depression/frontal cortex and hippocampus	Targeted metabolomics/LC-MS/MS	Alanine, aspartate, and glutamate pathways	[110]
	Fluoxetine hydrochloride	Depression patients/serum	Untargeted metabolomics/UPLC-Q-TOF-MS	Amino acid metabolism, energy metabolism, and lipid metabolism	[111]
	Ketamine	Treatment-resistant depression patients/plasma	Targeted metabolomics/LC-MS/MS and flow injection analysis-MS/MS	Lipid metabolism	[112]
	Ketamine	Mice (CVS)/hippocampus and prefrontal cortex	Untargeted Metabolomics/UPLC-Q-Orbitrap/MS	Sphingolipids, glycerolipids, and fatty acyls	[113]
	Ketamine	Humans/plasma and CSF, mice/plasma and brain	Targeted metabolomics/LC-MS/MS	LAT1, IDO1, NAD^+^, the nitric oxide (NO) signaling pathway, and sphingolipid rheostat	[114]
Traditional Chinese medicine	Bupleurum Chinense DC-Paeonia Lactiflora Pall Herb Pair	Rats (CUMS)/cortex	Untargeted metabolomics/UPLC-Q-Orbitrap/MS and targeted metabolomics/UPLC-MS/MS	Purine metabolism	[42]
	Chaigui Granules	Rats (CUMS)/peripheral blood mononuclear cell	Untargeted metabolomics/UPLC-Q-Orbitrap/MS	Purine metabolism	[115]
	Xiaoyao San	Rats (CUMS)/hippocampus	Untargeted metabolomics/UPLC-Q-Orbitrap/MS	Glucose catabolism	[116]
	Xiaoyao San	Rats (CUMS)/hippocampus	Untargeted metabolomics/GC-MS	D-glutamine and D-glutamate metabolism, arginine biosynthesis and alanine, aspartate, and glutamate metabolism	[117]
	Xiaoyao San	Depressed patients/plasma	Untargeted metabolomics/GC-MS	Oxalic and stearic acids	[118]
	Xiaoyao San	Rats (CUMS)/liver	Untargeted metabolomics/UHPLC-Q-Orbitrap/MS	Glutamine, glutamate, and energy metabolism	[119]
	Xiaoyao Pills	Rats (CUMS)/feces, brain, and plasma	Untargeted metabolomics/GC-MS	Metabolites from gut microbiota (benzoic acid, liquiritigenin, glycyrrhetinic acid, and saikogenin D) and fatty acids amide Hydrolase	[120]
	Jia Wei Xiao Yao San	Mice (CRS)/brain	Untargeted metabolomics/LC-TOF-MS and GC-MS	Purine metabolism	[121]
	Crocetin	Mice (CUMS)/serum, tissues, and feces	Targeted metabolomics/UPLC-Q-TOF/MS	Intestinal flora and tryptophan metabolism	[122]
	Schisandrin	Mice (LPS)/feces	Targeted metabolomics/GC-MS/MS	Short chain fatty acid	[123]
	Tongxieyaofang polysaccharide	Mice (CUS)/colon microflora	Untargeted metabolomics/UPLC-Q-TOF-MS	Bacterial community and bile acid metabolism	[124]
	Morinda officinalis oligosaccharides	Rats (CUMS)/plasma, brain, and feces	Targeted metabolomics/HPLC-MS/MS	Gut microbiota, serotonin, and 5-hydroxytryptophan	[47]
	Chaihu-Shugan-San	Mice (CUMS)/serum and liver	Targeted metabolomics/UHPLC- MS/MS	Gut microbiota, bile acids hyocholic acid, and 7-ketodeoxycholic acid	[125]
	Zhi-Zi-Chi decoctions	Rats (CUMS)/cecal contents, ileum, and hippocampus	Targeted metabolomics/LC-MS/MS and UHPLC-Q-TOF/MS	Butyrate	[126]
	Banxia Xiexin decoction	Atherosclerosis co-depression Mice/hippocampus and prefrontal cortex tissues	Untargeted metabolomics/UPLC-Q-Orbitrap/MS	Glycerophospholipid metabolism, lysophosphatidylcholine, and LPC (20:4) (rep)	[127]
	Paeoniflorin	Rats (CUMS)/urine	Untargeted metabolomics/UPLC-Q-Orbitrap/MS	Citrate cycle	[128]
	Jiaotaiwan	Rats (CUMS)/serum	Untargeted Metabolomics/UPLC-Q-TOF/MS	Amino acid, glycerophospholipid, and energy metabolism	[30]
	Albiflorin	Mice (CUMS, OBX, and LPS)/hippocampus	Targeted metabolomics/UPLC-MS/MS	Phospholipid and tryptophan metabolism	[129]
	Xiang-Su Volatile Oil	Menopausal rats by ovariectomy (CUMS)/plasma	Untargeted metabolomics/GC-MS	Phenylalanine, tyrosine, and tryptophan biosynthesis, tyrosine, and tryptophan metabolism	[130]
	Huang-lian Jie-du Decoction	Mice (CUMS)/hippocampus, cortex, striatum, and amygdala	Targeted metabolomics/LC-MS/MS	Tryptophan metabolism	[131]
	Berberine	Mice (CUMS)/hippocampus, prefrontal cortex, striatum, and amygdala tissues	Untargeted metabolomics/UPLC-Q-TOF/MS and targeted metabolomics/LC-MS/MS	Tryptophan metabolism	[132]
	Berberine	Rats (CUMS)/feces	Targeted metabolomics/GC-MS	Short chain fatty acids and monoamine neurotransmitters	[133]
	Quercetin	Rats (CUMS)/liver	Untargeted metabolomics/UPLC-MS	Methionine metabolism, bile acid metabolism, and phosphatidylcholine biosynthesis	[134]
	Acanthopanax senticosus	Mice (CUMS)/liver	Untargeted metabolomics/GC-MS	Glycine, serine, threonine, starch, and sucrose metabolism	[135]
	Radix Bupleuri-Radix Paeoniae Alba	Rats (CUMS)/serum	Untargeted metabolomics/UPLC-Q-Orbitrap/MS	Energy, amino acid, and lipid metabolism	[136]
	Bupleurum chinense DC-Paeonia lactiflora Pall	Rats (CUMS)/serum	Untargeted metabolomics/UPLC-Q-Orbitrap/MS	Saikogenin F and benzoic acid	[137]
	Baihe-Dihuang Tang	Rats (CUMS)/brain	Untargeted metabolomics/UPLC-Q-TOF/MS and targeted metabolomics/LC-MS/MS	l-glutamate, xanthine, and adenine	[138]
Other	l-Theanine	Rats (CUMS)/serum and hippocampal	Untargeted metabolomics/UPLC-Q-TOF-MS and targeted metabolomics/HILC-MS/MS	Amino acid metabolism and lipid metabolism	[139]
	Ferulic acid and feruloylated oligosaccharides	Mice (LPS)/serum	Untargeted metabolomics/UPLC-Q-Orbitrap/MS	Phenylalanine, tyrosine, and tryptophan biosynthesis, phenylalanine, and caffeine metabolism	[140]
	Dl-3-n-butylphthalide	Mice (CSDS)/brain	Targeted metabolomics/LC-MS/MS	Energy metabolism	[141]
	Edaravone	Mice (CSDS)/hippocampal and medial prefrontal cortex	Targeted metabolomics/LC-MS/MS	Energy metabolism	[142]
	Bifid triple viable capsule	Rats (CUMS)/serum and hippocampal	Untargeted metabolomics/UPLC-Q-TOF-MS	Biosynthesis of unsaturated fatty acids, glycerophospholipid, linoleic acid, and arachidonic acid metabolism	[143]
	Bifidobacterium breve CCFM1025	MDD patients/serum and feces	Targeted metabolomics/UHPLC-MS/MS	Gut microbiome and tryptophan metabolism	[144]
	Akkermansia muciniphila	Mice (CRS)/serum	Untargeted metabolomics/UHPLC-Q-Orbitrap MS	Hormone and neurotransmitter	[145]
	Lactobacillus	Depression mice induced by Ampicillin/cecum content	Targeted metabolomics/GC -MS/MS	Short-chain fatty acids	[146]
	Rifaximin	Rats (CUMS)/hippocampus	Targeted metabolomics/LC -MS/MS	Tryptophan metabolism	[147]
	Bacillus coagulans Unique IS-2	Rats (CUMS)/plasma	Targeted metabolomics/UPLC-Q-TOF-MS	l-Tryptophan, l-kynurenine, kynurenic-acid, 3-hydroxyanthranilic acid, acetate, propionate, and butyrate	[148]
	Aerobic exercise	Rats (CUMS)/serum	Untargeted metabolomics/UPLC-Q-Orbitrap/MS	Amino acid and energy metabolism	[149]
	Electroconvulsive therapy	Depressed patients/plasma	Targeted metabolomics/LC-MS	Tryptophan and kynurenine metabolites	[62]
	Repetitive transcranial magnetic stimulation	Mice (CUMS)/stool, plasma, prefrontal cortex, and hippocampus	Targeted metabolomics/GC-MS	Polyunsaturated fatty acids	[150]
	Repetitive transcranial magnetic stimulation	Treatment-resistant depression patients/plasma	Targeted metabolomics/LC-MS	Kynurenine metabolites	[61]

Abbreviations: major depressive disorder (MDD), chronic unpredictable mild stress (CUMS), chronic restraint stress (CRS), chronic unpredictable stress (CUS), chronic social defeat stress (CSDS), chronic variable stress (CVS), olfactory bulbectomy (OBX), lipopolysaccharide (LPS).

## Data Availability

Not applicable.
